# Natural Anti-biofilm Agents: Strategies to Control Biofilm-Forming Pathogens

**DOI:** 10.3389/fmicb.2020.566325

**Published:** 2020-10-29

**Authors:** Rojita Mishra, Amrita Kumari Panda, Surajit De Mandal, Muhammad Shakeel, Satpal Singh Bisht, Junaid Khan

**Affiliations:** ^1^Department of Botany, Polasara Science College, Polasara, India; ^2^Department of Biotechnology, Sant Gahira Guru University, Ambikapur, India; ^3^Key Laboratory of Bio-Pesticide Innovation and Application of Guangdong Province, College of Agriculture, South China Agricultural University, Guangzhou, China; ^4^Department of Zoology, Kumaun University, Nainital, India; ^5^Department of Pharmacy, Sant Gahira Guru University, Ambikapur, India

**Keywords:** microbial biofilm, therapeutic strategies, phytocompounds, multidrug resistance, antimicrobial peptides, biosurfactant

## Abstract

Pathogenic microorganisms and their chronic pathogenicity are significant concerns in biomedical research. Biofilm-linked persistent infections are not easy to treat due to resident multidrug-resistant microbes. Low efficiency of various treatments and *in vivo* toxicity of available antibiotics drive the researchers toward the discovery of many effective natural anti-biofilm agents. Natural extracts and natural product-based anti-biofilm agents are more efficient than the chemically synthesized counterparts with lesser side effects. The present review primarily focuses on various natural anti-biofilm agents, i.e., phytochemicals, biosurfactants, antimicrobial peptides, and microbial enzymes along with their sources, mechanism of action via interfering in the quorum-sensing pathways, disruption of extracellular polymeric substance, adhesion mechanism, and their inhibitory concentrations existing in literature so far. This study provides a better understanding that a particular natural anti-biofilm molecule exhibits a different mode of actions and biofilm inhibitory activity against more than one pathogenic species. This information can be exploited further to improve the therapeutic strategy by a combination of more than one natural anti-biofilm compounds from diverse sources.

## Background

The antimicrobial tolerance of biofilms has emerged as a significant challenge to medical scientists across diverse healthcare sectors. Synthetic drugs, combinational therapy, and antibiotic hybrids could not achieve and deliver the desired results during the treatment. The hunt for novel antimicrobials in drug resistance emergency insists on the scientific society to search novel natural anti-biofilm agents. The focus of the present review is to revisit various natural products to overcome the biofilm-forming microorganisms and provide concise information on existing confines and recent developments in the modification of different natural anti-biofilm agents to make them effective drug candidates for clinical exploitation.

### The Biofilms

The concept of biofilm was first developed by Marshall et al. (1971) and further described by Fletcher, Characklis, and Costerton, “Biofilm is the unique pattern of growth in the life cycle of microbes that provides specific properties, advantages and higher level of organization to the free living bacterial cells during colonization” (Characklis, 1973; Fletcher and Floodgate, 1973; Geesey et al., 1978; Flemming and Wuertz, 2019). The description of biofilm is much more clarified by Flemming and Wuertz (2019) that biofilms are aggregates of microorganisms with distinct sessile cells followed by cell division to form small clusters, microcolonies, and larger sums. The film underneath the biofilm is only in direct contact with the substratum in a multilayered heterogeneous microbial mat. Biofilms are extensively used in various biotechnological applications, for example, biofuel production, degradation of wastewater, and filtration of drinking water (Flemming et al., 2016b). The negative impact of biofilm includes bio-fouling (Flemming, 2011), corrosion (Little and Lee, 2015), and deterioration of the drinking water quality (Wingender and Flemming, 2011). All higher eukaryotes, including humans, are populated by microorganisms that form biofilms (De Vos, 2015). Human dental plaque, skin, and gut represent one of the dominant biofilms in eukaryotic habitats. The widespread uses of medical devices create several new niches for bacterial biofilm formations (Qvortrup et al., 2019).

Cells in biofilm survive harsh growth conditions as biofilms are surrounded by high molecular weight extracellular polymeric substances (EPS) that attach cells (Branda et al., 2005; Flemming and Wingender, 2010). The EPS are composed of proteins, lipids, polysaccharides, and extracellular DNA and play an essential function in the pathogenesis of the numerous microbial infections (Ch’ng et al., 2019). It has also been reported that microbial cells inside the biofilms are found to be resistant against UV, metal toxicity, acid exposure, desiccation, pH gradients, etc. (Costerton et al., 1999; Hall-Stoodley et al., 2004). In accretion to various physical and chemical tolerances, EPS confers immune resistance to many resident pathogenic microbes within biofilms by inhibiting neutrophil-mediated phagocytosis (Gunn et al., 2016). Izano et al. (2009) reported that the eDNA and intercellular adhesins of EPS act as a barrier for the penetration of a variety of antimicrobials. The eDNA present within the EPS chelate human antimicrobial peptides (AMPs) and lessen the antimicrobial activity of these peptides (Jones et al., 2013). So far, many studies have been carried out to identify the method of biofilm formation and subsequent preventive strategies to strike the challenges, especially the drug resistance due to biofilm formation (Roy et al., 2018).

**FIGURE 1:**
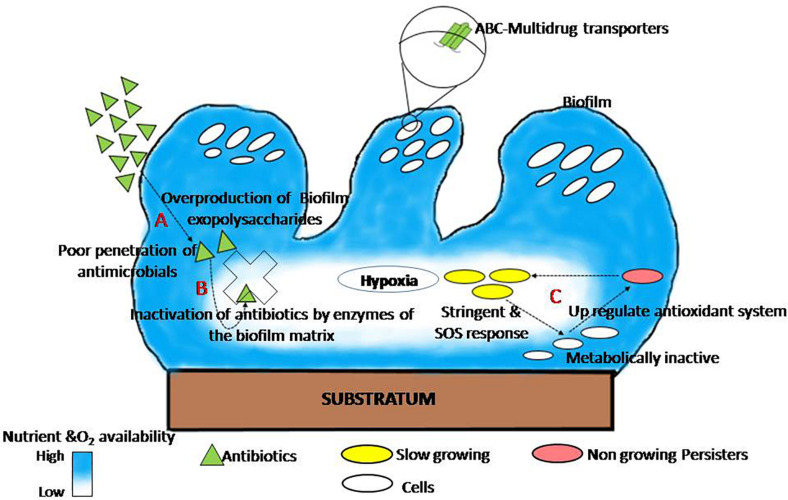
The general mechanism of biofilm tolerance to various antimicrobials. **(A)** Physical tolerance: biofilm matrix limits the diffusion of antimicrobials (Tseng et al., 2013). **(B)** Passive tolerance: matrix enzymes inactivate the penetrated antibiotics molecules (Fux et al., 2005). **(C)** Physiological tolerance: persister cells in the deeper layer of biofilm induce adaptive SOS response and thus become more tolerant (adapted from Ciofu and Tolker-Nielsen, 2019).

The presence of glycocalyx, outer membrane structure, and efflux pumps; and heterogeneity in growth rate, genetic adaptation, metabolic state, and metabolism of cells within a biofilm are the leading causes of biofilm that acquire resistance against antimicrobials (Singh et al., 2017). The mode of biofilm establishment in several human pathogens, as well as its drug resistance mechanism, is well documented and reviewed by different researchers and plotted (Figure 1). This figure explains the shared mechanism of biofilm tolerance under three sections. (1) Physical tolerance: the excess production of EPS restricts the penetration and diffusion of antimicrobials; as a result, cells in the biofilm get more time to become tolerant. Similar observations found that EPS production augments antimicrobial tolerance; an isogenic Δ*csgD* mutant of *Salmonella Typhimurium* (EPS-deficient mutant) is much susceptible to hydrogen peroxide (MacKenzie et al., 2017) and ciprofloxacin (Tabak et al., 2009). Therefore, therapeutic strategies that destabilize and inhibit EPS are the best anti-biofilm approaches to inhibit biofilms and reduce significant problems of antimicrobial resistance. A similar observation has been recorded by Deokar and Kadam (2020) and Dieltjens et al. (2020) that EPS inhibition reduces cell adhesion as well as drug tolerance in biofilms. (2) Passive tolerance: enzymes present in the biofilm matrix inactivate the antimicrobial molecules. The mechanism for the neutralization of antimicrobials through the biofilm matrix components have also been reported (Fux et al., 2005), and there are reports that catalase enzymes present in the biofilm matrix are responsible for tolerance of *Staphylococcus epidermidis* biofilm against various physicochemical agents (Olwal et al., 2019). (3) Physiological tolerance: metabolically inactive cells in the deeper layers of biofilm exhibit adaptive stress responses that regulate the tolerance of biofilms to various antimicrobials. Persister cells become more tolerant of a variety of antibiotics after phenotypic and reversible changes induced by starvation, ecological factors, and several other adaptive responses such as SOS and stringent response (Harms et al., 2016; Ciofu and Tolker-Nielsen, 2019; Soares et al., 2019).

Persister cells are slow-dividing bacteria, less susceptible to antibiotics, and they have an essential role for biofilm re-establishment (Dawson et al., 2011). Persister cells upregulate the expression of various toxin–antitoxin genes that blocks translation which leads to lessen cellular metabolism and eventually guarantees their survival in the presence of antibiotics (Lewis, 2005). These cells revive to vegetative dividing cells to reoccur infection after the end of antibiotic action (Lewis, 2010). Persister cells have been confined from antibiotics because these cells express the toxin-antitoxin system where the antibiotic target is blocked by the toxin module (Lewis, 2005). Oxygen scarcity and little metabolic activity in biofilms provide *P. aeruginosa* greater tolerance to many antibiotics (Wilkins et al., 2014). Sudden changes in pH between layers in a biofilm play a role to accumulate organic acids, thus deactivating the penetrating compounds (Wilkins et al., 2014). The development of gradients of oxygen, pH, nutrients, and electron acceptors all over the biofilm makes microenvironments where cells respond by altering their gene expression (Spormann, 2008; Stewart and Franklin, 2008). Complex (polymicrobial) biofilm made up of many species is generally more resistant to antibiotics than biofilm made up of a single species (Van der Veen and Abee, 2011). Cell diversity and metabolic conditions play the most important role in antibiotic tolerance of biofilms.

However, with the advance of sequencing technology, a ton of genomic data have been generated, which allows further illustration of the unknown molecular mechanism in the association of biofilm formation and drug resistance. The RNA-seq transcriptome analysis identified arsenic resistance operon genes (*arsR* and *arsD*), sporulation regulatory gene (*paiA*), ABC drug transporter classes, and penicillin-binding proteins associated with the *Enterococcus faecalis* biofilm formation and drug resistance (Seneviratne et al., 2017). They found higher-level expression of *arsD* in biofilm mode to avoid cell toxicity and suggested that *arsD* gene knockout could be a possible way of inhibiting biofilm formation. Similarly, they also observed the reduced level of expression of the *paiA* gene in biofilms. Padhi et al. (2016) reported that *Mtb* Rv0024 protein expression plays a significant role in the biofilm formation and subsequent resistance against anti-tuberculosis drugs in non-pathogenic *Mycobacterium smegmatis* strain.

The present tendency of antifungal tolerance is also a significant area of concern; therefore, direct research in a direction of novel antifungal compounds with targeted mechanisms of action are required (Sharma and Bisht, 2020). Many studies reported that the biofilm of *Candida albicans* is tolerant to many antifungal drugs as compared with the planktonic yeast cells. Moreover, the formation of mannan–glucan complex promoted by the extracellular vesicles (EVs) is also linked to drug resistance and reported in *C. albicans*, *C. glabrata*, *C. tropicalis*, and *C. parapsilosis* (Mitchell et al., 2015; Dominguez et al., 2018; Zarnowski et al., 2018). It has also been reported that overexpression of efflux pumps, a mutation in the target site of the drug, persisted cells, the interaction between biofilm and host immunity system as well as proteins associated with the filamentation process were involved in the biofilm-associated resistance mechanism of fungi (Borghi et al., 2016). Analyzing the transcriptional network regulating biofilm growth of *C. albicans* illustrates six major transcription regulators such as *Efg1* and *Tec1* for cell morphology regulation; *Bcr1*, *Brg1*, and *Ndt80* for biofilm formation; and *Tec1* and *Rob1* which controls the normal process of biofilm formation (Fox and Nobile, 2012; Uppuluri et al., 2018). Thus, an in-depth understanding of the molecular pathway involved in the biofilm formation and subsequent antibiotic resistance is essential to formulate the preventive measures. This review aims to focus on the natural anti-biofilm agents effective against a broad range of microbial biofilms and strategies related to recent biofilm treatments.

### Anti-biofilm Agents Based on Natural Products

**FIGURE 2:**
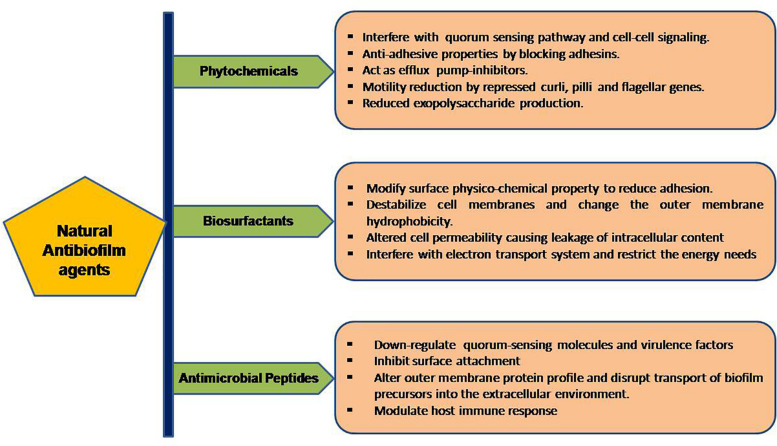
Workflow of the portrayed natural anti-biofilm agents based on their mode of actions.

The formation and development of biofilms is a complicated procedure involving different stages which can be the target of natural anti-biofilm agents for the prevention of biofilm development. Some of the well-studied stages of biofilm development include (1) attachment of bacterial cells to a suitable biotic/abiotic surface, (2) development of biofilm structure, (3) maturation of biofilm, and (4) dispersion (Boles and Horswill, 2008). The first two stages are highly critical in the development of biofilms and targeting one or both of these stages seems to be the ideal strategy for inhibition of biofilm formation. The attachment stage involves cytoskeletal elements (predominantly flagella, fimbriae) and lipopolysaccharides as key players. Surface signaling/communication of a group of bacteria, also termed as Quorum Sensing is a key player in the formation of biofilm. The natural anti-biofilm agents either act solely or synergistically by diverse mechanisms, as illustrated in Figure 2.

### Phytochemicals

There are broadly five classes of natural compounds that have high anti-biofilm properties. Those are phenolics, essential oils, terpenoids, lectins, alkaloids, polypeptides, and polyacetylenes (Yong et al., 2019). Phenolics are a group of compounds. It has seven subclasses which include phenolic acids, quinones, flavonoids, flavones, flavonols, tannins, and coumarins, out of which tannins, specifically condensed tannins, have anti-biofilm activity (Trentin et al., 2011). These entire compounds act on biofilm by six main mechanisms like substrate deprivation, membrane disruption, and binding to adhesin complex and cell wall; bind to proteins; interact with eukaryotic DNA; and block viral fusion (Cowan, 1999; Lu et al., 2019).

Several solvents, i.e., water, methanol, ethanol, chloroform, ether, dichloromethanol, and acetone, were used for the extraction of natural compounds from various sources for anti-biofilm activity. Various experiments carried out by researchers found that water extracts anthocyanins, sugars like tannins, saponins, terpenoids, polypeptides, and lectins. Ethanol extracts compounds, i.e., tannins, polyphenols, polyacetylenes, flavonol, terpenoids, sterols, alkaloids, and propolis whereas methanol extracts anthocyanins, terpenoids, saponins, tannins, xanthoxyllines, quassinoids, totarol, flavones, lactones, phenones, and polyphenols (Cowan, 1999). Extraction with chloroform yields terpenoids and flavonoids; dichloromethanol yields only terpenoids (Cowan, 1999); ethers when used as solvent results in the extraction of terpenoids, alkaloids, fatty acids, and coumarins whereas acetone isolates flavonols. Hydroquinone and caffeic acid methyl ester, isolated from *Cnestis ferruginea* Vahl ex DC. aqueous extract, showed promising results against *S. aureus* (Kouakou et al., 2019). Many researchers worked on bioactive compounds from medicinal plants for the discovery of novel natural anti-biofilm compounds. The anti-biofilm properties of Indian medicinal plants have been exploited and found that *Cinnamomum glaucescens* (Nees) Hand.-Mazz, *Syzygium praecox* Roxb. Rathakr. & N. C. Nair, *Bischofia javanica* Blume, *Elaeocarpus serratus* L., *Smilax zeylanica* L., *Acacia pennata* (L.) Willd., *Trema orientalis* (L.) Blume, *Acacia pennata* (L.) Willd., *Holigarna caustica* (Dennst.) Oken, *Murraya paniculata* (L.) Jack, and *Pterygota alata* (Roxb.) R. Br. extracts have promising anti-biofilm activity against *S. aureus* (Panda et al., 2020). 12-Methoxy-trans-carnosic acid and carnosol identified from the methanolic extract of *Salvia officinalis* L., an Algerian medicinal plant, have shown anti-biofilm activity against *Candida* biofilm in *in vitro* conditions (Kerkoub et al., 2018).

Phytochemicals inhibit the quorum sensing mechanism mainly by blocking the quorum sensing inducers like AHL, autoinducers, and autoinducers type 2 (Ciric et al., 2019). Garlic extracts play a vital role in the inhibition of quorum sensing signaling molecules of *Pseudomonas* and *Vibrio* spp. biofilms (Harjai et al., 2010; Lu et al., 2019). Emodin helps in the proteolysis of transcription factors associated with the quorum sensing and acts as its potent inhibitor (Ding et al., 2011). Quorum quenchers, along with antibiotics, are the best alternative anti-biofilm agents, as discussed by many researchers (Paluch et al., 2020). Phytochemicals also play a significant role in inhibiting bacterial adhesions and suppression of genes related to biofilm formation (Adnan et al., 2020). Biofilm development at the initial stages can be outlawed by interfering with the forces (Van der Waals force of attraction, electrostatic attraction, sedimentation and Brownian movements) which are responsible for the support of bacterial attachment to various surfaces (Roy et al., 2018). Phytocompounds have the potential to interfere with the extension along with the capability to stop the accessibility to nutrients essential for adhesion and bacterial growth (Sandasi et al., 2010).

There are reports on the anti-adhesive properties of ethanolic and acetone extract of *Psidium guajava* L. (Razak and Rahim, 2003) and extracts from various *Eugenia* spp. on *C. albicans* (Sardi et al., 2017). An alkaloid (norbgugaine) has shown a significant effect on *P. aeruginosa* biofilm by preventing adhesion due to loss of cell motility (Majik et al., 2013). A very recent study on *Adiantum philippense* L. crude extract has shown a promising role in decreasing the content of biofilm exopolysaccharides (Adnan et al., 2020). They observed that *Adiantum philippense* L. crude extract restrains biofilm at the initial stages by targeting adhesin proteins, deforming the pre-formed biofilms, and obstructing EPS production. Various researchers identified a different group of phytocompounds especially polyphenols such as 7-epiclusianone, tannic acid, and casbane which prevent cell surface attachment (Murata et al., 2010; Carneiro et al., 2011; Payne et al., 2013; Adnan et al., 2020).

Members of Enterobacteriaceae express curli, an amyloid fiber on the cell surface that helps in the attachment to characters and cell aggregation and enhances biofilm formation as well as cellular invasion (Tursi et al., 2020). Cegelski et al. (2009) found that pyridones affect the expression of the *CsgA* curli subunit and hamper curli biogenesis. Phloretin, ginkgolic acid, and phytocompounds from Malaysian plants help in the regulation of curli and pilli genes (Lee et al., 2011, 2014a, b; Johari et al., 2020). Vikram et al. (2013) reported that a citrus sterol β-sitosterol glucoside inhibited *E*. *coli* O157:H7 biofilm formation and motility by suppressing flagellar operon *flhDC*. The phytocompounds of curlicide and pilicide nature can be exploited in therapeutic strategies of Enterobacteriaceae biofilm prevention (Johari et al., 2020). Phytocompounds having fewer side effects can be a better therapeutic agent for biofilm-related infections, but recent reports suggest a combined approach which is always better than the individualistic approach. Few plant-based antimicrobials with the potential of anti-biofilm activity are summarized in Table 1.

**TABLE 1:** Anti-biofilm activity of phytocompounds with their mechanism of action.

Compound	Source	Experimental details	Pathogenic species	Molecular mechanism	Inhibitory concentration	References
Ajoene	*Allium sativum* L.	*In vitro* (PMNs killing assays) and *in vivo* (pulmonary infection mice model)	*Pseudomonas aeruginosa* *P. aeruginosa* *Staphylococcus* *aureus*	Downregulates rhamnolipid production Inhibits small regulatory RNA molecules (*rsmY*, *rsmZ*, and *rnaIII*) that operate in the later phase of QS signaling	20 μg/ml ajoene reduces rhamnolipid production by 1/3 IC_50_ for *rsmY* = 2.5 μg/ml *rsmZ* = 2.3 μg/ml	Jakobsen et al., 2012 Jakobsen et al., 2017
Allicin	*Allium sativum* L.	*In vitro* (Δ*pqsABCD* knockout strain)	*Pseudomonas aeruginosa*	Decreases the bacterial adhesion in the initial stages of biofilm formation as it reduces EPS formation It controls the expression of virulence factors hence interfere with the QS system	250 μM inhibit production of virulence factors such as pyocyanin, elastase, and pyoverdine and rhamnolipids	Xu et al., 2019
Carvacrol (monoterpenoid)	*Origanum vulgare* L.	*In vitro* (qPCR for relative expression of *lasI*/*lasR* genes) and docking modeling of proteins LasI and LasR	*Pseudomonas aeruginosa*	Post-translational inhibition against *lasI*, which effects AHL production. It mainly acts on QS machinery	C6-AHL production reduced up to 80% with 1.9 mM of carvacrol	Tapia-Rodriguez et al., 2019
Emodin (anthraquinone)	*Polygonum cuspidatum* Siebold & Zucc. and *Rheum palmatum* L.	*In vitro* (crystal violet biofilm assay and SEM analysis)	*Staphylococcus aureus*	Decreases the release of eDNA and downregulates the expression of biofilm-forming related genes like *cidA*, *icaA*, *dltB*, *agrA*, *sortaseA*, and *sarA*	MIC = 8 μg/ml	Yan et al., 2017
Emodin (anthroquinone)	*Rheum palmatum* L.	*In vitro* (microdilution assay, kinase assay) and molecular docking for emodin in CK2 (Autodock Vina)	*Candida albicans* *Candida krusei* *Candida parapsilosis* *Candida tropicalis*	Biofilm formation is inhibited by targeting cellular kinase signaling It acts on planktonic cells by reducing hyphal formation. It acts as a competitive inhibitor of CK2	MIC = 12.5 μg/ml MFC = 25 μg/ml MIC and MFC = 25 μg/ml MIC and MFC = 50 μg/ml	Janeczko et al., 2017
Aloe-emodin	*Rheum officinale* Baill.	*In vitro* (CLSM assays and Congo red assay)	*Staphylococcus aureus*	Reduce the production of extracellular proteins and polysaccharide intercellular adhesin	Inhibited biofilm formation on polyvinyl chloride surfaces at 32 μg/ml	Xiang et al., 2017
Hordenine	*Hordeum vulgare* L. (sprouting)	*In vitro* (SEM and CLSM assays, qPCR for QS-related genes)	*Pseudomonas aeruginosa*	Decreases AHL production Virulence factors (proteases, elastase, pyocyanin, rhamnolipid, alginate, and pyroviridine) production decreased significantly. Inhibit swimming and swarming activity Down-regulates the expression of *lasI, lasR, rhlI* and *rhlR* genes.	1 mg/ml of hordenine along with 0.4 μg/ml of netilmicin reduced biofilms by 88% C4-HSL production decreased up to 69% at 0.5 mg/ml	Zhou et al., 2018
Pulverulentone A	*Callistemon citrinus* (Curtis) skeels leaves	*In vitro* (broth microdilution assay, CLSM, TEM analysis)	Methicillin-resistant *Staphylococcus aureus*	Reduces styphyloxanthin production, thus inhibiting biofilm formation Disrupts the cell membrane	MIC = 125 μg/ml Production of the virulence factor decreased by 65.9%	Shehabeldine et al., 2020
Vitexin (flavon)	*Vitex species*	*In vitro* (safranin staining, microscopy methods, EPS quantification) *In vivo* murine model (catheter-associated infection), molecular docking	*Pseudomonas aeruginosa*	Attenuates formation of EPS, QS-associated factors (swarming motility, production of protease, pyoverdin and pyocyanin) Molecular docking studies confirmed it attnuates *Las A*, *Las B*, and *Lux R*	MIC = 260 μg/ml 39.04% decrease in Las A protease and 37.54% Las B elastase	Das et al., 2016
5-Hydroxymethylfurfural	*Musa acuminata* Colla.	*In vitro* (biofilm, Las B elastase, protease, and rhamnolipid quantification assays)	*Pseudomonas aeruginosa*	Inhibits the production of biofilm proteins, EPS, and cell surface hydrophobicity productions Downregulates the expression of QS-regulated virulence genes	MBIC = 400 μg/ml Reduces production of biofilm proteins, biofilm adherence, EPS and CSH to the level of 79, 82, and 77%, respectively Inhibits the production of *LasA* protease, *LasB* elastase, pyocyanin, alginate, and rhamnolipid 77, 75, 68, 80, 78, and 69%, respectively	Vijayakumar and Ramanathan, 2020
Phytol	*Piper betle* L.	*In vitro* (microscopic analysis, transcriptional analysis of QS-regulated genes)	*Serratia marcescens*	Inhibits the swarming motility and hydrophobicity Downregulates QS genes	Significantly inhibits the production of biofilm and EPS to the level of 65 and 43%	Srinivasan et al., 2016
Isolimonic acid and ichangin	*Citrus* species	*In vitro* (Caco-2 cell adhesion and survival assay, AI-3 reporter assay)	Enterohaemorrhagic *Escherichia coli* *Vibrio harveyi*	Decreases the adherence Downregulates flagellar genes, *ler* (transcriptional regulator of LEE) Represses the expression of the flagellar master regulator (*flhC* and *flhD*) Regulates *luxO* expression, thus acting as potent modulators of bacterial cell–cell signaling	IC_25_ (isolimonic acid) = 19.7 μM IC_25_ (ichangin) = 28.3 μM ler repressed by 5-fold, *flhC* and *flhD* repressed by 4.6 and 6.9, respectively IC_50_ (isolimonic acid) = 94.18 μM	Vikram et al., 2012 Vikram et al., 2011
(R)-Bgugaine	*Arisarum vulgare* O. Targ. Tozz.	*In vitro* (static biofilm inhibition assay)	*Pseudomonas* *aeruginosa*	Affects flagella related functions, inhibits pyocyanin pigmentation, LasA protease, rhamnolipid production.	Reduces biofilm density by 83% at 1.8 mM	Majik et al., 2013
Zingerone	*Zingiber officinale* Roscoe	*In vitro* (microtiter plate assay, motility assay, quorum sensing signal molecules quantitative assay) and molecular docking of TraR, LasR, and PqsR proteins	*Pseudomonas aeruginosa* PAO1	Reduces swimming, swarming, and twitching motility. Suppresses pyocyanin, hemolysin, rhamnolipid, protease and elastase Molecular docking analysis proved that it could bind with all the quorum sensing receptors and stops receptor–ligand interaction, suppresses QS-dependent gene expression	Sub MIC = 10 mg/ml Reduces the BFC of *P. aeruginosa* PAO1 as A_570_ from 1.1 to 0.5	Kumar et al., 2015
Baicalin	*Scutellaria baicalensis* Georgi	*In vivo* (mouse peritoneal implant infection model)	*Pseudomonas aeruginosa*	Inhibits *LasA* protease, *LasB* elastase, pyocyanin, rhamnolipid, motilities and exotoxin A virulence factors Decreases the expression of *lasI*, *lasR*, *rhlI*, *rhlR*, *pqsR*, and *pqsA* genes and reduces the QS signaling molecule 3-oxo-C12-HSL and C4-HSL	MIC > 1024 μg/ml C4-HSL levels decreased 77.2% at 64 μg/ml baicalin	Luo et al., 2017
Curcumin	*Curcuma longa* L.	*In vitro* (crystal violet biofilm assay, pellicle formation assay, surface motility assay, mixed culture biofilm assay) *In vivo* (*Caenorhabditis elegans* model organism, *C. elegans* killing assay)	*Acinetobacter baumannii*, *C. albicans*	Inhibits pellicle formation, Pilli motility, and ring biofilm formation Molecular docking analysis proved that curcumin interacts with the biofilm response regulator BfmR	MIC > 500 μg/ml for *A. baumannii* ATCC 17978 planktonic cell Reduces *A. baumannii* ATCC 17978 biofilm production by 93% at 100 μg/ml	Raorane et al., 2019
Epigallocatechin-3-gallate (EGCG)	*Camellia sinesis* (L.) Kuntze (green tea)	*In vitro* (growth assay, CR-binding assay, TEM analysis)	*Escherichia coli* BW25113	Suppresses curli production and expression of curli-related proteins *csgA*, *csgB*, and *csgD* Enhances the degradation of sigma factor (RpoS) by ClpXP protease	IC_50_ = 5.9 ± 0.8 μM	Arita-Morioka et al., 2018
Ginkgolic acid (GA) and hydroginkgolic acid	*Pistacia lentiscus* L. (fruit)	*In vitro, in vivo* (human lung A549 infection model, *C. elegans* infection model)	*Pseudomonas aeruginosa* H103	Decreases virulence factor production Modifies the membrane fluidity Regulates virulence through the ECFσSigX	IC_50_ of pyocyanin inhibition = 6.3 μg/ml 100 μg/ml GA reduces pyocyanin production by 82%	Tahrioui et al., 2020
7-Epiclusianone	*Rheedia brasiliensis* (Mart.) Planch. & Triana	*In vivo* A rodent model of dental caries	*Streptococcus mutans*	Increases cariostatic activity by disrupting insoluble exopolysaccharides and intracellular polysaccharides	70–80% less severe smooth-surface lesions and 50–70% less severe sulcal-surface lesions than the vehicle control treatment 50–70% reduction of exopolysaccharides	Murata et al., 2010 Branco-de-Almeida et al., 2011
Tannic acid	Not specified	*In vitro* (crystal violet microplate biofilm assay, Congo red binding assay)	*E. coli* BW25113	Efficiently killed bacteria in *pgaA* mutant biofilms by inhibiting the formation of polysaccharide in the matrix Affects intracellular SOS response and decreases the expression of genes involved in this pathway	MIC = 1 mg/ml	Samoilova et al., 2019a Samoilova et al., 2019b
Diterpene derivative (C_31_H_50_O_3_)	*Myrmecodia pendens* Merr. & L.M. Perry	*In vitro* (broth microdilution assay, MBIC analysis by Perumal method)	*Streptococcus mutans* ATCC 25175	Not specified	MBIC = 50 ppm and MIC = 40 ppm	Gartika et al., 2018
Chelerythrine	*Bocconia cordata* Willd.	*In vitro* (broth microdilution assay, crystal violet assay) and *in vivo* (mono- and dual species culture models)	*Candida albicans* and *Staphylococcus aureus*	Inhibits hyphae formation Reduces biofilm formation by decreasing eDNA, polysaccharide, and protein levels	The MICs (monospecies) = 4 μg/ml and MBIC_90S_ (monospecies) = 2 μg/ml MICs (dual species) = 6 μg/ml and MBIC_90S_ (dual species) = 3 μg/ml	Qian et al., 2020
Hyperforin	*Hypericum perforatum* L.	*In vitro* (quorum sensing inhibition assay, human plasma protein-coated assay, static microtiter plate crystal violet assay)	*Staphylococcus aureus* AH1872	Exhibits anti-biofilm activity and a moderate amount of quroum quenching activity, but a detailed mechanism is not specified	MIC_50_ (flowering aerial part) = 0.512% *v*/*v* Exhibit moderate inhibition of quorum sensing (QSIC_50_ = 0.064–0.512% *v*/*v*)	Lyles et al., 2017
Warburganal, polygodial, alpha-linolenic acid (ALA)	*Warburgia ugandensis* Sprague subsp. *ugandensis*	*In vitro* (tetrazolium reduction assay, checkerboard assay)	*Candida albicans* *Candida glabrata* *S. epidermidis* *S. aureus*	α,β-unsaturated 1,4-dialdehyde in polygodial and warburganal is responsible for the potent antifungal activity on developing biofilms Polygodial affects mitochondrial ATPase and leads to reduced ergosterol levels	BIC_50_ (warburganal) = 4.5 ± 1 μg/ml and BIC_50_ (polygodial) 10.8 ± 5 μg/ml BIC_50_ (warburganal) = 37.9 ± 8 μg/ml BIC_50_ (ALA) = 25 μg/ml	Kipanga et al., 2020

*NS, not specified.*

### Biosurfactants

Biosurfactants (BS) hinder biofilm formation by varying the cell adhesion ability through less cell surface hydrophobicity, membrane disruption, and inhibited electron transport chain, thus restricting cellular energy demand (Satpute et al., 2016). Biosurfactants of different classes are produced by various microorganisms that exhibit antibacterial, antifungal, and anti-biofilm activities (Paraszkiewicz et al., 2019). The effect of biosurfactants from *Lactobacillus plantarum* and *Pediococcus acidilactici* on quorum sensing signaling molecules and expressions of biofilm-linked genes in *Staphylococcus aureus* was evaluated (Yan et al., 2019). They reported that biosurfactants reduce the growth of *S. aureus* biofilm by regulating the expression of biofilm-related genes *dltB*, *icaA*, *cidA*, etc. BS from *Lactobacillus plantarum* significantly reduced cidA gene expression at 12.5 mg/ml (Yan et al., 2019). Biosurfactant from *Pediococcus acidilactici* downregulates the gene expression of autoinducer-2 (AI-2) signaling molecules, accessory gene regulator (*agr A*), and staphylococcal accessory regulatory (sar A) at 50 mg/ml (Yan et al., 2019). Previous studies reported that the anti-biofilm activity of *Lactobacillus*-derived BS loaded liposomes had greater ability than free BS to inhibit *S. aureus* (MRSA) biofilm formation and elimination (Giordani et al., 2019). Few biosurfactants along with their consequence on biofilm growth, development, and dispersal are summarized in Table 2.

**TABLE 2:** Biosurfactants reported recently with anti-biofilm activities.

Class	Source microorganism	Pathogen strains	Effect on biofilm	Dose	References
Lipopeptide biosurfactants (LPBs)	*Acinetobacter junii*	Biofilm of *Staphylococcus aureus*, *Proteus mirabilis*, and *Pseudomonas aeruginosa*	Biofilm disruption 35, 10, and 32%, respectively Biofilm disruption 52, 31, and 70%, respectively	1250 μg/ml 2500 μg/ml	Ohadi et al., 2020
Lipopeptide	*Beauveria bassiana*	*Microsporum canis*	25.76% biofilm eradication	1.95 μg/ml	Abdel-Aziz et al., 2020
Lipopeptide surfactin-C15	*B. subtilis* #309	*Candida albicans*	85% inhibition to biofilm formation	960 μg/ml	Janek et al., 2020
Lipopeptide surfactin	*Bacillus safensis* F4	*Staphylococcus epidermidis*	80% anti-adhesive activity	6.25 mg/ml	Abdelli et al., 2019
Lipopeptide pontifactin	*Pontibacter korlensis* strain SBK-47	*Bacillus subtilis*, *Staphylococcus aureus*, *Salmonella typhi*, and *Vibrio cholerae*	99% anti-adhesive activity	2 mg/ml	Balan et al., 2016
Lipopeptide	*Bacillus subtilis* AC7	*Candida albicans*	Reduced adhesion up to 67–69% and biofilm formation up to 56–57%	2 mg/ml	Ceresa et al., 2016
Glycolipoprotein	*Acinetobacter indicus* M6	Methicillin-resistant *Staphylococcus aureus*	82.5% removal of biofilm	500 μg/ml	Karlapudi et al., 2020
Glycolipid	*Burkholderia* sp. WYAT7	*Staphylococcus aureus*	41% inhibition to biofilm formation 79% inhibition to biofilm formation	1 mg/ml 2 mg/ml	Ashitha et al., 2020
Rhamnolipids	*Pseudomonas aeruginosa* MN1	*Streptococcus mutans*	Dissociation of 67% of the preformed biofilm	12.5 mg/ml	Abdollahi et al., 2020
Rhamnolipids	*Burkholderia thailandensis* E264	*Streptococcus oralis*, *Actinomyces naeslundii*, *Neisseria mucosa*, and *Streptococcus sanguinis*	90% inhibition of *S. sanguinis* biofilm 70% inhibition of *S. oralis* biofilm 70% inhibition of *N. mucosa* biofilm 50% inhibition of *A. naeslundii* biofilm	0.39 mg/ml 0.78 mg/ml 6.25 mg/ml 12.5 mg/ml	Elshikh et al., 2017
Exopolysaccharides	*Pandorea pnomenusa* MS5	*Burkholderia cepacia*	Inhibit *Burkholderia cepacia* biofilm formation	0.25 mg/ml	Sacco et al., 2019

Ohadi et al. (2020) identified an anionic lipopeptide from *Acinetobacter junii* which self-aggregates to form β sheet–rich biosurfactant vesicles. This biosurfactant is thermostable and less toxic, so it can be used as an anti-biofilm agent. Biofilms that are developed by dermatophytes are very difficult to eradicate. A lipopeptide biosurfactant obtained from *Beauveria bassiana*, which is an insect-attacking fungus, plays an important role as an anti-biofilm agent in *ex vivo* conditions for *M. canis* (Abdel-Aziz et al., 2020). It acts by disrupting cell membrane integrity and interfering with cell membrane permeability. The biosurfactant from *B. bassiana* overcome the disadvantage of expensive production as it was produced from steep corn liquor. This can be a promising biosurfactant for recalcitrant dermatophytosis. Surfactin, a cyclic lipopeptide, was found to be very effective along with its metal complex against *C. albicans* biofilm-related infections. This biosurfactant also controls the expression of hyphal specific genes and mainly act by decreasing cellular surface hydrophobicity (Janek et al., 2020).

Rhamnolipids produced from *Pseudomonas aeruginosa* MN1 have higher anti-adhesive and anti-biofilm activity than that of surfactin (Abdollahi et al., 2020). Glycolipid isolated from *Burkholderia* sp. WYAT7, an endophyte of *Artemisia nilagirica* (Clarke) Pamp, has anti-biofilm activity against *S. aureus* (Ashitha et al., 2020). A broad-range glycolipoprotein, rich in Leu-His-Trp amino acids identified from *Acinetobacter indicus* M6 has low toxicity and removed 82.5% of biofilm at a concentration of 500 μg/ml (Karlapudi et al., 2020). The EPS from *Pandoraea pnomenusa* MS5 serves as an anti-biofilm agent against *Burkholderia cepacia* (Sacco et al., 2019). This surfactant is a heteropolysaccharide, with two functional carbonyls and hydroxyl groups, and has oil-emulsifying capacity.

Biosurfactants are appropriate coating agents for medical implants such as urinal catheters, bone implants, etc. to inhibit biofilms originated from pathogenic organisms without using synthetic drugs. Rhamnolipids and sorphorolipids are reported to be potential agents for the inhibition of biofilms formed by Gram-negative and Gram-positive microbes (Sharahi et al., 2019). Few studies reported that cell-associated biosurfactant from *Lactobacillus acidophilus* inhibits biofilm formation of *Proteus vulgaris* and *S. aureus* on polydimethylsiloxane (PDMS)-based implants (Satpute et al., 2019). *L. rhamnosus*–derived biosurfactants cause cell lysis by disrupting the membrane structure, thus can be used as an anti-biofilm agent for multispecies biofilms on silicone devices, i.e., voice prostheses in case of laryngectomy (Tan et al., 2017). The anti-biofilm activity of biosurfactants can augment extensively in combination with caprylic acid that inhibits biofilm formation of *P. aeruginosa*, *E. coli*, and *B. subtilis* (Diaz De Rienzo et al., 2016); amphotericin B (AmB) or fluconazole synergistically acts against biofilm formation and preformed biofilm of *C. albicans* (Haque et al., 2016); and surfactants such as SDS led to the destruction of *P. aeruginosa* PAO1 biofilms (Nguyen et al., 2020).

### Antimicrobial Peptides

AMPs are broad-acting antimicrobial agents widely used in the treatment of both fungal and bacterial biofilms (Pletzer et al., 2016). These peptides disrupt biofilms developed on medical devices such as catheters, artificial valves, stents, dentures, etc. occupied in hospital-acquired infections by *S. aureus*, *Klebsiella pneumoniae*, *P. aeruginosa*, *Enterococcus faecium*, *Acinetobacter*, and *Enterobacter* spp. (ESKAPE), and non-ESKAPE pathogens (Rajput and Kumar, 2018). AMPs are substitute to traditional antibiotics that are less vulnerable to bacterial resistance by attacking the bacterial cell membrane (Hirt et al., 2018). AMPs occur naturally in humans, animals, plants, and microbes and act on bacterial cell membranes by interacting with membrane phospholipids electrostatically, followed by insertion into membrane, thus killing bacteria. There are reports of synergizing AMPs with antimicrobial compounds to suppress various molecular pathways of biofilm formation (Shahrour et al., 2019).

Amphibian skin is a source for many AMPs effective against various biofilm-causing microorganisms. Yuan et al. (2019) isolated an AMP Japonicin-2LF from Fujian large-headed frog skin secretion (*Limnonectes fujianensis*) that inhibits MRSA biofilms by membrane permeabilization. Japonicin-2LF behaves like a detergent and eradicates both planktonic and sessile pathogens in biofilms. This property can be exploited to use this peptide as a promising drug candidate in cystic fibrosis patients for the cure of MRSA infection. The main drawback of using AMPs to treat biofilm-based infections is that they are very much prone to degradation by various bacterial proteases.

An AMP from frog skin named esculentin-1a, i.e., Esc (1-21), and its D-amino acid–containing diastereomer Esc (1-21)-1c inhibited *P. aeruginosa* biofilm formation by its membrane-perturbing activity. Previous studies reported that Esc (1-21)-1c showed potential activity against chronic lung *Pseudomonas* infections of cystic fibrosis patients (Casciaro et al., 2019). The introduction of D-amino acids at Leu^14^ and Ser^17^ into the AMP (esculentin-1a) sequence increases the AMP stability (Casciaro et al., 2019); decreases *P. aeruginosa* swimming, swarming, and twitching motility; and finally inhibits biofilm formation. The peptide inhibits the *P. aeruginosa* biofilm formation by three mechanisms. First, it downregulates the *fleN* gene that controls the number of flagella in *P. aeruginosa* inhibiting flagella-mediated swimming. Second, it decreases the mRNA level of type IV pili biosynthesis genes at very low concentration, i.e., 1/8 MIC, and inhibits the twitching motility of *P. aeruginosa* that is very much essential for micro-colony formation and colonization during biofilm development. Third, it downregulates *lasI* gene encoding for the quorum-sensing molecule acyl-homoserine lactone (AHL) synthase as well as *lasB* gene encoding the virulence factors elastase *LasB*.

In summary, Esc (1-21)-1c lowers the expression of virulence genes and bacterial motility genes, and ultimately prevents biofilm formation. These two anti-pseudomonal peptides esculentin-1a (1-21) and its diastereomer Esc (1-21)-1c have shown promising results in bronchial epithelium repair of cystic fibrosis patients. Cappiello et al. (2019) observed that esculentin repairs bronchial epithelium in cystic fibrosis patients by promoting bronchial cell migration, activating epidermal growth factor receptors, and also increases the secretion of IL-8 for the re-epithelialization process. Besides, the peptide esculentin-1a lowers the expression of mRNA encoding rhamnosyltransferase subunits, i.e., *RhlA* and *RhlB*, key enzymes in the biosynthesis of bacterial surfactant rhamnolipids. A recent study by Parducho et al. (2020) reported a specific mode of action of the AMP human Beta-Defensin 2 that increases the roughness of the bacterial surface, alters outer membrane protein profile, and interferes with the transfer of biofilm precursors into the extracellular space (Figure 2).

The melittin peptide of bee venom exhibit antibacterial activity, prevents MRSA systemic infections and initiates the wound healing process in MRSA-infected mice model (Choi et al., 2015). Khozani et al. (2019) studied the efficiency of melittin and found that it degraded about 90–95% of *P. aeruginosa* biofilm biomass at 50 μg concentrations during 24 h. Human cathelicidin LL-37 inhibits bacterial adhesion and biofilm mass of *S. epidermidis* ATCC 35984 at a very low concentration (Hell et al., 2010). In addition to anti-biofilm activity, LL-37 exhibits immunomodulatory activity such as cellular recruitment (Tjabringa et al., 2006), enhances host adaptive immune responses (Diamond et al., 2009), and modulates inflammatory responses (Mookherjee et al., 2006). The dual property of AMPs to counter bacterial biofilms as well as modulating the host immune system can be exploited to design a novel strategy to combat drug-resistant microbial biofilms.

Parducho et al. (2020) reported the inhibitory property of human Beta-Defensin 2 in *P. aeruginosa* biofilm by inducing structural changes, altering outer membrane protein profile and interfering with the transfer of biofilm precursors into the extracellular space. Few natural peptides with anti-biofilm activity and their disadvantages are listed in Table 3. A peptide DRAMP ID: DRAMP18417 derived from the venom of scorpion (*Tityus obscurus*) has shown promising anti-biofilm activity against *Candida* spp. and *Cryptococcus neoformans* strains (Guilhelmelli et al., 2016). These peptides inhibit fungal biofilms at initial adhesion and mature stages, and exhibit minimal hemolytic and cytotoxic activity on erythrocytes and murine peritoneal macrophages.

**TABLE 3:** Sources and effects of AMPs.

Name of AMPs	Amino acid sequence	Net charge	3D structure	Source	Effects on biofilm	Disadvantages	References
Japonicin-2LF	FIVPSIFLLK KAFCIALKKC	4	Helix	Frog skin secretion	Eradicates the methicillin-resistant **S. aureus** biofilm matrix as well as kills all the sessile bacteria	Futile against *P. aeruginosa* biofilms. The anti-biofilm activity concealed by the changes in LPS contents and cell wall structure of microorganisms	Yuan et al., 2019
Dermaseptin-PT9	GLWSKIKDAAKT AGKAALGFVNEMV	2	Helix	Frog skin secretion	Inhibits the biofilm formation of **S. aureus**, MRSA, and **E. coli**	More potent activity against Gram-negative bacteria	Li et al., 2019
Phylloseptin-PTa	FLSLIPAA ISAVSALANHF	2	Helix	Frog skin secretion	More potent against **S. aureus** biofilm	Anti-biofilm activity changed by the hydrophobicity, charges and α-helicity of the peptides	Liu et al., 2017
Moronecidin-like	FFRNLWKGAK AAFRAGHAAWRA	6	Unknown	Seahorse	Inhibits surface attachment of *S. aureus* biofilm	More effectual against Gram-positive bacteria than Gram-negative bacteria The outer membrane proteins of Gram-negative bacteria may hinder translocation of AMPs through the outer membrane	Mohammadi et al., 2018
Mastoporan	LNLKALL AVAKKIL	4	Helix	European hornet venom	Suppresses biofilm formation by *S. aureus* and *P. aeruginosa*	Release histamine from mammalian mast cells may lead to an immune response	Chen et al., 2018
Melittin	GIGAVLKVLTTG LPALISWIKRKRQQ	6	Helix	Honeybee venom	Induce disintegration of the MDR *P. aeruginosa* and degrades the biofilm	The toxicity of melittin on normal cells is a disadvantage for clinical applications (in case of third-degree burn patients, all three layers of skin are destroyed, so cytotoxicity of melittin hardly limits its applications)	Khozani et al., 2019
NA-CATH	KRFKKFFKKLKNSV KKRAKKFFKKPKVIGVTFPF	15	Helix	Chinese cobra (*Naja atra*)	Prevent biofilm formation of *Burkholderia thailandensis*	The small size of the peptide restricts its large-scale synthesis	Blower et al., 2015 de Barros et al., 2019
Defensin ZmD32	RTCQSQSHRFRGPCLRRS NCANVCRTEGFPGG RCRGFRRRCFCTTHC	12	Combine Helix and Beta structure	Corn, **Zea mays**	Active against *Candida albicans* biofilms	Anti-biofilm activity of many defensins lost in the presence of salt	Kerenga et al., 2019
Capsicumicine	RSCQQQIQQ AQQLSSCQQYLKQ	–	Unknown	Red pepper, **Capsicum bacattum**	Prevents the establishment of *S. epidermidis* biofilm by matrix anti-assembly (MAA) mechanism	NS	Von Borowski et al., 2020
Rhesus theta defensin-1	GFCRCLCRRGVCRCICTR	5	Beta	Monkey leukocytes	Active against established *C. albicans* biofilms	Most of the host defense peptides exhibit undesirable pro-inflammatory properties and low bioavailability	Basso et al., 2019

*NS, not specified.*

Pathogens which form the biofilms on the implanted medical devices, human skin, gut, and oral cavities generally communicate through quorum sensing (QS) signals. The quorum sensing inhibiting potential of AMPs from natural sources offers an alternative antibiotic-free approach to overcome biofilm-associated infections. To date, more than 3000 AMPs have been discovered, but only seven of them have been approved by the FDA (Chen and Lu, 2020). There is a severe scarcity of clinical studies on natural AMPs due to their poor performance, cytotoxic and hemolytic activities, unpredicted side effects such as kidney injuries, damage to central nervous systems, etc. The futility of natural AMPs in pre-clinical stages may be due to variations between the clinical setting and their resident conditions. So, clinical research needs to be exaggerated and optimized for the use of these natural anti-biofilm agents against various drug-tolerant biofilms. It is essential to exploit the structure of different naturally occurring AMPs to develop novel therapeutic peptides with improved stability and activity in comparison with their natural counterparts.

Efforts have been made to design novel specifically targeted multi-domain AMPs composed of a species-targeting peptide linked to a broad-spectrum antimicrobial killing peptide domain (Sztukowska et al., 2019). C16G2 is one of the first functional specifically targeted AMPs designed by fusion of a 16−mer region of the *Streptococcus mutans* competence−stimulating peptide (CSP) as the targeting domain, flexible triglycine linker and G2 AMP, a 16−residue fragment of novispirin G10 (Steinstraesser et al., 2002), and a derivative of ovispirin-1 (N-terminal 18 residues of the sheep cathelicidin SMAP29). C16G2 inhibits the biofilm growth of *S. mutans* effectively both in pure culture and in a multispecies community (Guo et al., 2015). This peptide not only kills *S. mutans* but also reduces other species that are metabolically dependent on *S. mutans* and mediates the re-establishment of oral microbiome. This specifically targeted AMP avoids the loss of natural microflora by selectively targeting the pathogens and leaving commensal *Streptococci* undamaged. C16G2 has completed a single-blind, open-label phase II clinical trial in various varnish and strip formulations (ClinicalTrials.gov Identifier: NCT03196219) among female and male dental subjects.

Similarly, attempts have been made to target only the pathogenic organisms of the biofilm without influencing the normal microflora (Xu et al., 2020). They designed peptides by fusing species-specific enterococcal pheromone cCF10 with a broad-spectrum AMP C6. They proved that incorporation of cCF10 at the N terminus of C6 drastically increased antimicrobial activity against *E. faecalis* comparative with C6 alone. They also reported that the hybrid peptides stimulated negligible hemolysis against human RBCs at antimicrobial levels, demonstrating that these fusion peptides could be exploited as potential anti-biofilm agents for clinical implementation.

### Therapeutic Strategies Using Natural Products

**FIGURE 3:**
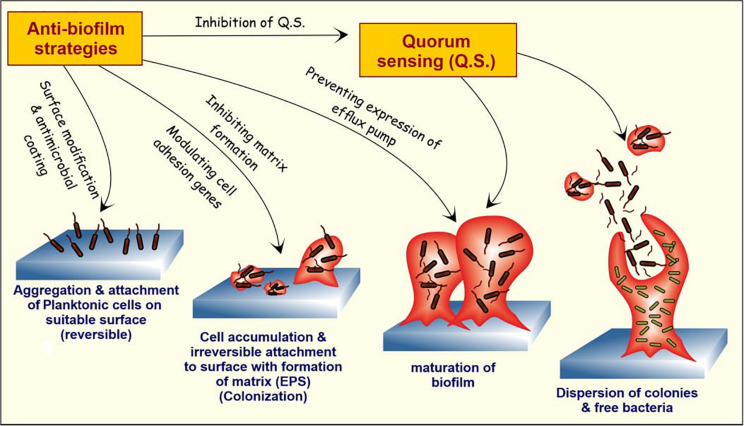
The stages of biofilm formation and potential targets for anti-biofilm agents. The bacterial cells in humans attach to the matrix-forming proteins by forming a covalent linkage with peptidoglycan structure or by non-covalent attachment. With attachment and aggregation of a sufficient number of cells, the formation of EPS matrix takes place, and the attachment now becomes resistant to external repulsive forces. With the maturation of biofilm, the cells within the bulk structure start further communication with each other and start secreting specialized proteins and DNA, and some of them are involved in the formation of the efflux pump. At last, the dispersion of free planktonic cells from the formed biofilm further promotes the formation of new biofilms in the periphery. The natural anti-biofilm compounds can attack at one or different stages of biofilm formation and development, thus inhibiting it.

The failure of conventional antibiotic therapies indicates that biofilm treatments need auxiliary upgradation (Zhang et al., 2020). Natural anti-biofilm agents selectively exterminate the persistent biofilms and allow the diffusion of antimicrobials into the biofilm matrix. These natural products target various phases of biofilm cycle to degrade the biofilm matrix and finally kills the released cells (Figure 3). A better understanding of the disruption and dispersal mechanism of biofilms will help researchers to design improved anti-biofilm strategies.

**FIGURE 4:**
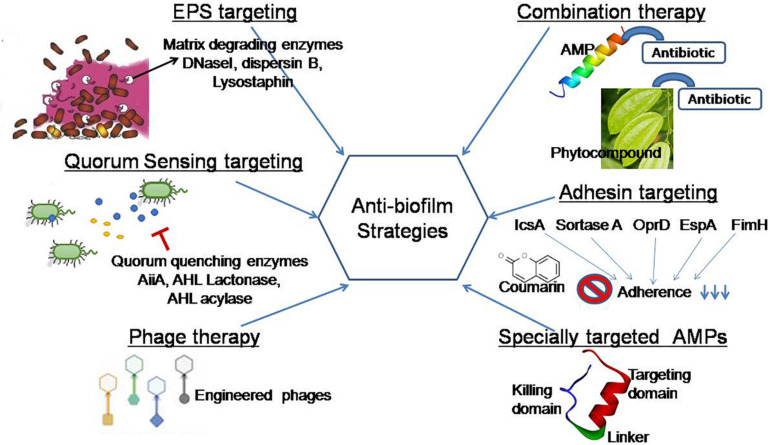
Graphical representation of anti-biofilm strategies covered in this review. EPS targeting: EPS matrix is targeted by matrix-degrading enzymes (DNaseI, dispersin B, lysostaphin) that inhibit microbial adhesion to a surface. Quorum sensing targeting: This strategy focused on the use of natural agents that block cell–cell communication in preformed biofilms and regulate virulence factor production (Shastry et al., 2019). Phage therapy: Engineered phages degrade the matrix exopolysaccharide by producing polysaccharide depolymerase enzymes. Specially targeted AMPs: This novel strategy targets in a species-specific manner due to the presence of species targeting peptides (Xu et al., 2020). Adhesin targeting: Phytocompounds target adhesin proteins and blocked biofilm formation at the beginning (Adnan et al., 2020). Combination therapy: Natural anti-biofilm agents function effectively in a combined approach in comparison with its single use.

A recent study reported that elasnin (an anti-biofilm compound from an actinobacteria *Streptomyces mobaraensis* DSM 40847) destroyed the matrix in a multispecies biofilm and making them more vulnerable to antibiotics (Long et al., 2020). To improve the current strategies of biofilm inhibition, the concern of the present review is to exploit natural agents for the development of an effective and safe strategy. This review aims to cover some current systems that are being put into practice to disintegrate EPS, quench QS networks, inhibit adhesion, and interrupt biofilm formation (Figure 4).

### Extracellular Polymeric Substance (EPS)–Targeting Strategies

Microbial EPSs secreted by a large variety of microorganisms mainly composed of polysaccharides, structural proteins, and extracellular DNA. The EPS matrix supports microbial adhesion to a surface, aggregation in multilayered biofilms, and functions as a three-dimensional scaffold that provides hydration, digestive capacity, and protection against antimicrobial compounds, antibiotics, and host effecter molecules (Flemming et al., 2016a). The EPS matrix can actively alter nutrient gradients and portray pathogenic environments that contribute to tolerance and virulence traits. So, many therapeutic strategies are designed to target the EPS matrix to eliminate biofilms, disaggregate bacteria, and interrupt the pathogenic environment. Many bacterial enzymes and secondary metabolites interfere with the quorum sensing mechanisms of pathogenic bacteria, thus disrupting the biofilm formation (Khan et al., 2019). Biofilm matrix-degrading enzymes such as beta-*N*-acetylglucosaminidase and dispersin B secreted by the Gram-negative periodontal pathogen *Actinobacillus actinomycetemcomitans* disintegrate mature biofilms of *Staphylococcus epidermidis*. Cocktail of two EPS-degrading enzymes, DNase I and dispersin B, has been found to inhibit staphylococcal skin colonization and remove pre-attached *S. aureus* cells from the skin and enhance their povidone-iodine susceptibility in an *in vivo* pig skin colonization model (Kaplan et al., 2018). Hogan et al. (2017) reported that lysostaphin was an effective anti-staphylococcal agent and its therapeutic efficacy can be improved in combination with antibiotics. Few biofilm-degrading enzymes with their mechanism of biofilm inhibition are summarized in Table 4.

**TABLE 4:** Biofilm-degrading enzymes against various human pathogens.

Enzymes	Source	Pathogenic bacteria	Molecular mechanism of biofilm inhibition	References
Serine protease, Esp	*Staphylococcus epidermidis*	*Staphylococcus aureus*	Esp degrades *S. aureus* surface proteins and host receptors	Sugimoto et al., 2013
Lysostaphin	*Staphylococcus simulans*	Methicillin-resistant *Staphylococcus aureus* (MRSA)	Cleaves the pentaglycine cross-bridges of peptidoglycan and destroyed EPS matrix	Algburi et al., 2017 Hogan et al., 2017
α-Amylase	*Bacillus subtilis* S8-18	Methicillin-resistant *Staphylococcus aureus* (MRSA)	Degrades the preform mature biofilm by disrupting EPS matrix	Kalpana et al., 2012
Cellulase	*Penicillium funiculosum* *Trichoderma reesei*	*Pseudomonas aeruginosa*	Decreases the adhesion of cells to the surface and polysaccharide matrix	Loiselle and Anderson, 2003
Cellulase	*Aspergillus niger* *Bacillus* sp. DGV19	*Burkholderia cepacia*	Degrades the exopolysaccharide	Rajasekharan and Ramesh, 2013
Alginate lyase	*Bacillus circulans* ATCC 15518	*Pseudomonas aeruginosa*	Degrades the exopolysaccharide	Alkawash et al., 2006
Hyaluronan	*Streptococcus equi*	*Staphylococcus aureus*	NS	Ibberson et al., 2016
Cysteine, histidine dependent amidohydrolase/peptidase CHAP_*K*_	Myoviridae staphylococcal Phage K	*Staphylococcus aureus*	Cleaves the peptide bond involving D-alanine and the first glycine in the pentaglycine cross-bridge of *Staphylococcal* cell wall peptidoglycan	Fenton et al., 2013
Endolysin LysH5	Phage vB_SauS-phiIPLA88	*Staphylococcus aureus*, *Staphylococcus epidermidis*	Anti-persister agents	Gutierrez et al., 2014
DNase I	Human stratum corneum	*Pseudomonas aeruginosa*, *Staphylococcus aureus*	Degradation of extracellular DNA prevents the formation of biofilm	Eckhart et al., 2007
DNase I and Proteinase K	NS	*Actinomyces oris*, *Fusobacterium nucleatum*, *Streptococcus mutans*, *Streptococcus oralis*, and *Candida albicans*	Affected the structural integrity of the biofilms by removal of eDNA and extracellular proteins	Karygianni et al., 2020
Trypsin	Pancreatic serine endoprotease	*Pseudomonas aeruginosa*	Destroy the protein contents of the biofilm matrix	Banar et al., 2016

*NS, not specified.*

The existing enzymes which have less catalytic activity can enhance their catalytic properties against the biofilms by modeling and engineering approach. The site-directed mutational analysis is considered as another approach to modulate the biofilm-inhibiting properties of the enzymes. Thus, broad-spectrum enzymes/peptides, as well as secondary compounds, must be isolated from bacteria for bioprospection, which can target a broad range of QS signaling molecules and structural part of the biofilms. The complete elimination of heterogeneous biofilms needs amalgamation of hydrolytic enzymes that can degrade proteins, polysaccharides, eDNA, and QS molecules (Yuan et al., 2020). The application of matrix-degrading enzymes in biofilm control is presently limited due to cost, handling procedures, and low industrial accessibility (Nahar et al., 2018).

### Quorum Sensing Targeting Strategies

Prevention of cell-to-cell communication (quorum sensing) is an efficient strategy to restrain biofilm formation (Sharahi et al., 2019). It is reported that metalloprotein AHL-lactonase from the cell-free extract of endophytic *Enterobacter* species causes degradation of *N*- AHL, thus significantly inhibiting biofilm formation by *Aeromonas hydrophila* (Shastry et al., 2019). The result of a recent study reported that *Lactobacillus crustorum* ZHG 2-1 as novel quorum-quenching bacteria degrade *N*-3-oxododecanoyl-*dl*-homoserine lactone (3-oxo-C12-HSL) and *N*-butyryl-*dl*-homoserine lactone (C4-HSL) and functions as an anti-biofilm agent against *P. aeruginosa* (Cui et al., 2020). Several quorum quenching (QQ) enzymes and compounds have been reported. The majority of these QQ molecules have been isolated from natural sources (LaSarre and Federle, 2013). The result of a recent study revealed QS inhibitory potentials of ethyl acetate extracts from cell-free supernatants and cells of *Natrinemaversi forme* against *P. aeruginosa* biofilm (Başaran et al., 2020). Many QS inhibitors from plant-based natural products have been identified (Caceres et al., 2020; Zhong et al., 2020) and proposed to be effective in future biofilm targeting strategies. The role of natural anti-biofilm agents in the inhibition of quorum sensing molecules is mentioned in the first part of the review. Here, we attempted to explain their effect on the disruption of QS mechanism. These anti-biofilm agents disrupt quorum-sensing systems mainly in two ways: (1) inhibition and degradation of signal molecules, and (2) mimicking the signal molecules for inhibition of their binding to corresponding receptors (Kalia, 2013).

On the other hand, quorum quenchers are usually species specific; therefore, a combination of quenchers is required to eliminate mixed-species biofilms. Ajoene, a sulfur-rich molecule from garlic, decreases the expression of small regulatory RNAs (sRNAs) in both Gram-negative (*P*. *aeruginosa*) and Gram-positive (*S*. *aureus*) bacteria. Ajoene is the first compound to be identified to target broad-spectrum range quorum sensing inhibitors, i.e., lowers the RNAIII expressions in *S*. *aureus* (Scoffone et al., 2019) and RsmY and RsmZ in *P*. *aeruginosa* (Jakobsen et al., 2017). Ajoene lowered expression of small regulatory RNAs (*rsmY* and *rsmZ*) in *P*. *aeruginosa* as a result of which it represses translation of biofilm matrix polysaccharides Pel and Psl and the type VI secretion system (T6SS). The T6SS in *P. aeruginosa* plays an essential role in the expression of various virulence factors and greatly concerned with the biofilm formation, pyocyanin production, and pathogenicity of the organism (Li et al., 2020). These findings suggest that T6SS may be a prospective therapeutic target against *P. aeruginosa* infections. Jakobsen et al. (2017) also found that ajoene lowers the expression of regulatory RNA and RNAIII, and inhibits the expression of RNAIII-dependent virulence factors such as lipase, protease, and α-hemolysinin in *S*. *aureus*. Emodin (1, 2, 8-trihydroxy-6-methyl anthraquinone), an anthraquinone derivative identified from *Rheum palmatum* (Chinese rhubarb) and *Polygonum cuspidatum* (Asian knotweed), effectively downregulated *luxS* gene in *Streptococcus suis* (Yang et al., 2015) and *icaA*, *sarA*, and *agrA* genes in *S. aureus* (Yan et al., 2017).

The anti-biofilm peptide Human Cathelicidin LL-37 affects the bacterial cell signaling system and inhibits *P. aeruginosa* biofilm formation at 0.5 μg/ml by downregulating genes of the QS system (Di Somma et al., 2020). AMPs interact with membranes of bacteria and, in turn, activate genes that are regulated through QS. These QS autoinducers passed through the plasma membrane with the help of membrane vesicles. This process, in turn, activates the expression of virulence genes associated with QS. Autoinducers help in interspecies signal transduction; one interesting autoinducer is small autoinducing peptide molecule (AIP) from *Lactobacilli* that inhibits the viability of microbes and acts as a suppressor of bacteriotoxin production. During the process of suppression of exotoxin production, they interfere with the agr QS system (Vasilchenko and Rogozhin, 2019). However, quorum quenchers can be rinsed away during biofilm formation that makes limited uses of these inhibitors confined to small areas of biofilm only (Koo et al., 2017). Thus, combination approach of these inhibitors along with other strategies leads to a novel therapeutic approach.

### Phage Therapy

Lytic bacteriophages have been used as an effective therapeutic strategy to remove biofilm cells. A recently published study proved that two lytic phages vB_SauM_ME18 and vB_SauM_ME126 are potential natural antimicrobials for inhibiting biofilm of MDR *S. aureus* (Gharieb et al., 2020). Recent investigations have shown that (engineered) phage-derived enzymes—polysaccharide depolymerase or peptidoglycan-degrading enzymes—are promising therapeutic anti-biofilm candidates (Reuter and Kruger, 2020). Phage therapy got its first FDA approval in the year 2019 in which patients received phage treatment at the School of Medicine, University of California San Diego (UCSD) phage therapy center (Pires et al., 2020). The administration of phage therapy is active only in a few countries, and its clinical use faces many challenges such as the establishment of phage banks with characterized phages; safety, stability, and quality of phage preparations during production; and the evolution of bacterial resistance to phages.

### Combination Therapy

Natural anti-biofilm agents sensitize antibiotics and established to be more effective when used in amalgamation (Zhang et al., 2020). They also reported that the combined application of sodium houttuyfonate and levofloxacin act in a better manner to inhibit biofilm formation. Sodium houttuyfonate, a plant-derived anti-neuropeptide, effectively disrupts biofilm dispersion in *P. aeruginosa* (Wang et al., 2019). Naringin, a flavanone glycoside extracted from citrus and grapefruits, was found to be more effective against *P. aeruginosa* biofilms in comparison with individual treatment of marketed antibiotics ciprofloxacin and tetracycline (Dey et al., 2020). Naringin depletes biofilm EPS and facilitates the diffusion of antimicrobials, reduces pellicle formation, and decreases the flagellar movement of bacteria on catheter surfaces.

Zhou et al. (2018) tested the effect of hordenine, a polyphenolic compound from barley, on biofilm formation individually as well as in combination with an aminoglycoside antibiotic, netilmicin. The results were promising, showing up to 88% reduction in *P. aeruginosa* PAO1 biofilms by a combination of hordenine and netilmicin, which was significantly better than the effect of any of the individual treatments. It indicates that the drug–herb combination therapy may be explored for effective anti-biofilm formulation opportunities. The SEM study showed a reduction in the thickness of the biofilm layer and the disruption of its architecture. The results of the study also revealed downregulation in the expression of quorum-sensing regulatory genes, especially *lasR*, by hordenine as the possible mechanism against biofilm development. Actinobacterial compounds from different microbial species have also shown potential anti-biofilm activity against different pathogenic bacteria by interrupting the cell surface and interaction between cells (Azman et al., 2019). Studies focusing on a combination of more than one natural anti-biofilm compound/s from different sources or acting on different stages of biofilm development will further help in developing more effective agents targeting biofilms. Moreover, the selection of a more effective compound is also necessary as the efficacy of natural compounds against biofilm development is different against different strains of bacteria.

### Anti-biofilm Biomaterial Therapy

The adhesion of biofilm-associated pathogenic organisms on implant surfaces restricts their clinical applications, so many attempts have been made by various researchers to coat biomaterial as a preventive strategy. Natural polymer-based surface coatings, such as anti-adhesive coatings of algal polysaccharide ulvan, dextran, and dermatan sulfate, and antimicrobial-releasing polysaccharide coatings etc. have been popularized during the last decade (Junter et al., 2016). A recent report on anti-adhesive CyanoCoating (a coating from marine cyanobacterium *Cyanothece* sp. CCY 0110) was exploited as a defensive strategy against a broader range of microbes (especially *Proteus mirabilis*, *E. coli*, and *C. albicans* biofilms) in catheter-linked urinary tract infections (Costa et al., 2020). The molecular mechanism to prevent the cell adhesion is that the hydrophilic polysaccharides form a hydration layer on the surface which acts as a physical barrier (Damodaran and Murthy, 2016) and prevents cell adhesion to the surface. Calcium phosphate cement and hydroxyapatite are the calcium phosphate materials that are used as a bone coating to avoid infections of biofilm, but they have various limitations in clinical trials (Pan et al., 2018). Implant-related infections can be avoided by chitosan hydrogel coatings which prevent bacterial adhesion and biofilm formation due to membrane leaching (Pan et al., 2018). A group of natural polymers were used as drug transporters in various forms like fibers, strips, gels (Badam gum, Karaya gum, chitosan), films (chitosan), nanoparticles, and microparticles which help in delivering antibiotics to the targeted site mainly for periodontal biofilm-forming pathogens (Chi et al., 2019). Nisin, an FDA-approved AMP, acts as anti-biofilm agent synergistically with conventional antibiotics against methicillin-resistant *Staphylococcus aureus*, *Streptococcus pneumoniae*, *Enterococci*, and *Clostridium difficile* (Shin et al., 2016). A recent report stated that nisin in conjugation with gellan gum, a biocompatible polysaccharide, shows promising results in biomaterial research (Peng et al., 2020).

## Conclusion and Future Directions

The occurrence of many biofilm-based human infections and their multiple antimicrobial resistance is a major concern in medicine and human health. The elevated rate of resistance to antibiotics in biofilm leads to the discovery and characterization of novel natural anti-biofilm agents. This review describes different types of phytocompounds, antimicrobial peptides, and biosurfactants that exhibit promising biofilm-inhibiting ability. Natural anti-biofilm agents could be effectively used to deal with certain surgeries and diseases where there is a possibility of untraceable infection sites like bone, dental, eye lenses, and breast implants. These agents of natural origin are structurally and functionally more diverse in comparison with conventional antibiotics. The structure and function of natural anti-biofilm agents from various sources have been exploited to develop numerous advanced therapeutic strategies showing increased activity, stability, and reliability. Here, we continue to analyze the efficacy of specially targeted AMPs against drug-tolerant pathogenic biofilms without disturbing the natural microflora.

Natural products, mainly phytochemicals, as anti-biofilm agents have been studied more in *in vitro* and *in vivo* conditions, but not a single FDA-approved drug was developed despite huge efforts. Most of them failed in phase II and phase III clinical trials (Lu et al., 2019). The possible reason for this failure may be the availability of the compound in humans after administration which decreases the efficacy of the compounds. One possible solution to this problem is a combination of strategies like antibiotics, along with natural anti-biofilm agents for better results. Combination therapy of natural agents with commercial antibiotics needs urgent exploitation in the future to advance anti-biofilm activity. Quorum quenchers of natural origin along with antibiotics can be a novel lead for species-specific biofilm destruction, and it has a promising utilization aspect in biomedical industries. More studies should be directed in this regard for converting the novel anti-biofilm phytocompounds into drugs. Most of the clinical studies on natural anti-biofilm compounds as reported in http://clinicaltrials.gov/are focused on oral biofilms, and very few are related to urinary tract infections (Lu et al., 2019). Further studies in *in vivo* models and clinical trials are needed to test the efficiency of natural anti-biofilm agents in the future.

The review also explains the quorum quenching molecules and EPS-degrading enzymes of natural origin along with their mode of action on various biofilms. The mechanism of action of various natural agents against biofilm remains unknown. More studies on the mode of action may help to identify novel anti-biofilm agents. Anti-adhesin strategy can be a novel strategy for biofilm treatments on a broad range of bacteria as it targets and prevents attachment of bacteria to the cell surface. Very few studies have been made in this area, so future research in targeting biofilm in the direction of adhesin proteins may lead to the discovery of unique natural anti-biofilm agents. Pili and curli gene expression regulating phytocompounds can control biofilm formation. More work in these directions or a combination of phytocompound which has anti-adhesin properties may be a better therapeutic strategy for biofilm-related ailments.

The failure of natural medicines in clinical trials can be checked by rigorous quality control. The discovery of accurate markers that are sensitive and stable can resolve the problem and help in better quality control of natural anti-biofilm agents. It is a significant challenge faced by natural product research for the discovery of useful QC markers as natural compounds have a very complex structural lattice (Zhang et al., 2020). Drug efficacy of natural compounds is mainly based on network pharmacology methods. As a result, more research in this direction can enhance success rate in clinical trials at the final stage. Novel natural anti-biofilm agents in therapeutics may be possible if rigorous studies will be done in quality control, pharmacokinetic and pharmacodynamic co-relationships (PK–PD), and PK–PD interactions with metabolomics of host for evaluation of safety and efficacy of the drug.

## Author Contributions

AP, RM, SD, MS, and SS drafted the manuscript. AP, RM, and JK were responsible for preparing the tables and figures in the manuscript. AP and RM equally contributed to the development of this manuscript. SS assisted to revise the manuscript. All the authors read and approved the final manuscript.

## Conflict of Interest

The authors declare that the research was conducted in the absence of any commercial or financial relationships that could be construed as a potential conflict of interest.

## Abbreviations

AHL: acyl-homoserine lactone
AMP: anti-microbial peptide
BFC: biofilm-forming capacity
CLSM: confocal laser scanning microscopy
CSH: cell surface hydrophobicity
eDNA: extracellular DNA
EPS: extracellular polymeric substances
GA: ginkgolic acid
HSL: homoserine lactone
MBIC: minimum biofilm inhibitory concentration
MFC: minimal fungicidal concentration
MIC: minimum inhibitory concentration
PMNs: polymorphonuclear leukocytes
QS: quorum sensing
SEM: scanning electron microscopy
TEM: transmission electron microscopy.
